# Discovery of a mcl-PHA with unexpected biotechnical properties: the marine environment of French Polynesia as a source for PHA-producing bacteria

**DOI:** 10.1186/s13568-015-0163-y

**Published:** 2015-11-25

**Authors:** P. Wecker, X. Moppert, C. Simon-Colin, B. Costa, V. Berteaux-Lecellier

**Affiliations:** LabEX Corail, USR3278 CNRS-EPHE-UPVD CRIOBE, BP1013, Papetoai, Moorea, French Polynesia; Pacific Biotech SAS, BP140289, Arue, Tahiti, French Polynesia; Laboratoire de Microbiologie des Environnements Extrêmes, LM2E, UMR6197 IFREMER-UBO-CNRS, BP 70, Plouzané, France

**Keywords:** Polyhydroxyalkanoate, Marine biotechnology, Bacterial diversity, French Polynesia, Fermentation

## Abstract

A library of microorganisms originating from various marine environments in French Polynesia was screened for polyhydroxyalkanoate producing bacteria. No significant connection was found between the geo-ecological source of bacteria and their ability to produce polyhydroxyalkanoate. A bacterial strain designated as *Enterobacter* FAK 1384 was isolated from a shark jaw. When grown on coprah oil, this bacterium produces a PHA constituting of 62 mol % 3-hydroxydecanoate and lower amount of 12 mol % 3-hydroxydodecenoate and of 7.6 mol % 3-hydroxydodecanoate. These interesting properties make this mcl-PHA a good candidate for further exploitations in many industrial sectors, as in film and coating manufacturing, as well as for biomedical applications.

**Electronic supplementary material:**

The online version of this article (doi:10.1186/s13568-015-0163-y) contains supplementary material, which is available to authorized users.

## Introduction

Microorganisms are widely recognized as a source of novel enzymes, biocatalysts and bioactive compounds, as well as biomaterials. Until recently, marine microorganism diversity was quite neglected in industrial processes in comparison to terrestrial species (Imhoff et al. 2011). In the last years, biotechnological interest in the exploitation of marine environments has been increasing and scientific literature is steadily producing new discoveries of bioactive marine metabolites (Imhoff et al. 2011). In particular, unusual environments with extreme conditions are a natural treasure chest for innovative bioactive molecules (Ferrer et al. 2005). Among these are bacterial biopolymers, and more particularly polyhydroxyalkanoates (PHA), which are biopolyesters produced by many microorganisms as intracellular storage material. It is noteworthy that their monomeric composition varies depending on the host and on the available carbon source. In addition, PHA comprise a large class of polyester. They can be separated into three groups, depending on the number of carbons atoms in the monomeric units: short-chain-length, medium-chain-length and long-chain-length PHA. The short-chain-length PHA (scl-PHA) are ubiquitous. They contain primarily 3-hydroxybutyrate (3 HB) units and show limits in their mechanical properties (Hazer and Steinbuechel 2007; Kim do et al. 2007; Samrot et al. 2011). In contrast, medium-chain-length PHA (mcl-PHA) are synthetized by only a few bacteria species belonging to four to five genera. The polymers produced consists mainly of 3-hydroxyoctanoate (3HO) and 3-hydroxydecanoate (3HD), and appear to be much more elastomeric and resistant (Solaiman et al. 2000; Verlinden et al. 2007; Samrot et al. 2011; Kabilan et al. 2012). The commercial processes for PHA production go way back into the 1960s. In addition, molecular genetic studies give valuable insights into the regulation of PHA formation since the 1990s (Madison and Huisman 1999). Biodegradable PHA substitute more and more petrochemical plastics due to their physical and structural properties (Rawte and Mavinkurve 2002). Unfortunately, the production costs remain the economical limitation. To achieve a cost-effective PHA production scheme, the isolation of new bacterial strains able to utilize inexpensive carbon sources has become a focus of particular interest (González-García et al. 2008).

The still largely unexploited marine environment specifically provides a new research area to isolate new microorganisms (Table 1). French Polynesia is a large territory, consisting of 118 islands forming five archipelagos, with a landmass of only 3500 km^2^ scattered over 5.5 million km^2^ of ocean. Polynesia has some particularities in this field owing to its geographical position and the presence of specific extreme ecosystems, such as microbial mats called ≪kopara mats/ponds≫ (Richert et al. 2005), uninhabited volcanic islands, hot springs, etc. The taxonomic inventory of the Polynesian island groups is still limited to the main islands of the Society, Tuamotu and Austral archipelagos, due to their geographical location (Lozouet et al. 2004). Only the major groups—corals, fishes, molluscs, echinoderms and algae—have received particular attention in the last years; knowledge of the other invertebrate groups and especially bacteria is still fragmentary, except the all-taxon microbial inventory of one Polynesian coral reef system (McCliment et al. 2012). This lack of knowledge and those different types of ecosystems make the marine environment of French Polynesia very interesting for biotechnological investigations, as is reflected already in various publications (Guezennec et al. 2011; Raguénès et al. 2004; Richert et al. 2005; Simon-Colin et al. 2008a, b, c, 2009).

**Table 1:** Selection of published articles dealing with PHA-producing bacteria from the marine environment

Source	Locations	Biopolymer	Isolates (References)
Marine sediment	Pakistan	mcl-PHA	*Pseudomonas* sp. CMG607w (Jamil et al. 2007)
Marine sediment		PHB	*Vibrio *spp. (Chien et al. 2007)
Decaying sea marsh grass	USA	PHA	*Saccharophagus degradans* ATCC42961 (González-García et al. 2008)
Kopara mats	French Polynesia	mcl-PHA	*Pseudomonas guezennei* sp. nov. (Simon-Colin et al. 2008a) *P. raguenesii* sp. nov. (Simon-Colin et al. 2009) *P. guezennei biovar* (Simon-Colin et al. 2008c)
Deep-sea hydrothermal vent shrimps	Mid-Atlantic Ridge	PHA	*Halomonas profundus* sp. nov. (Simon-Colin et al. 2008b)
Soil, marine mangrove sediments, back and sea water	India	PHB	*Vibrio* sp. MK4 (Arun et al. 2009)
Sea water	India	PHB	*H. hydrothermalis* SM-P-3M (Shrivastav et al. 2010b)
Sea water	India	PHA	*Spirulina subsalsa* (Shrivastav et al. 2010a)
Seawater	Japan	PHA	*Vibrio* sp. KN01 (Numata and Doi 2012)
Contamination in stock culture on marine agar plate		PHA	*Bacillus* sp. (Sawant et al. 2014)

Therefore, the aim of this study was the screening of a bacterial library originating from the marine biodiversity around French Polynesia for promising PHA-producing microorganisms and to get hints about geo-ecological preferences. In this study, we present a novel mcl-PHA with interesting mechanical and physical structural properties produced by a new strain *Enterobacter* sp. FAK 1384, isolated from a shark jaw (*Carcharhinus melanopterus*) nearby Fakarava Island (Tuamotu, French Polynesia).

## Material and methods

### Collection, isolation, cultivation and phylogenetic identification of PHA-producing bacteria

Since 2001, samples were collected from different geomorphological places over four of the five archipelagos of French Polynesia, which include marine sediment, marine animals, microbial mats, microbial films and the water column. Each sample was placed in a Zobell medium overnight. The resulting colonies were isolated and purified on Zobell marine agar medium using serial dilution technique. The plates were maintained in aerobic condition at 32 °C for 24–48 h. Each pure isolate was then cultivated and placed into a Nunc^®^ cryotube at minus 80 °C with glycerol (20 %) as cryoprotector.

Screening of marine bacterial PHA producers was performed in nitrogen-free Zobell medium enriched with glucose at 20 g/L. The Nile Red coloration method (Spiekermann et al. 1999) and the Sudan Black staining method (Schlegel et al. 1970) were used together to reveal the lipophilic compounds of PHA-producing bacteria. Pure isolates containing lipophilic inclusions were identified based on microscopy and by sequencing partial sequences of their 16SrRNA. Cultures were sent to Macrogen (http://www.macrogen.com) for DNA isolation and sequencing. The results were compared with the 16S rRNA sequence available in public nucleotide databases at the National Center for Biotechnology Information (NCBI) by using its World Wide Web site (http://www.ncbi.nlm.nih.gov) and the basic local alignment search tool (BLAST) algorithm (Altschul et al. 1990). Multiple sequence alignment and phylogenic analysis were performed by the neighbour-joining (NJ) method embedded in the Mega 5 software (Tamura et al. 2011). The sequence of the 16S rRNA encoding gene of strain FAK 1384 was determined and deposited to GenBank [GenBank: KJ499995].

### PHA production

Production of PHA polymers was accomplished in two steps. The first one consisted of the biomass production in rich marine medium. The PHA production took place in the second step, in a nitrogen-free medium with glucose or coprah oil as carbon source. Both steps were performed in shake flask cultures (250 mL). The growth step took place for 16 h at 32 °C and 250 rpm agitation, in a rich medium (peptone 8 g/L, yeast extract 2 g/L, glucose 10 g/L and synthetic sea salt 10–30 g/L according to the strain). The medium was then centrifuged (8000×*g* for 10 min) and the cell pellets were transferred into another flask containing sea salt (10–30 g/L) and glucose or coprah oil under the same conditions. After 48 h, cells were centrifuged (10 min at 20,000×*g*) and cell pellets were lyophilized.

Strain designated as FAK 1384 showed interesting PHA production in flask and was further cultivated in a 5 L bioreactor (Sartorius, Biostat A plus). Temperature was maintained at 32 °C, pH at 7.6 by automatic addition of 2 mol/L NaOH and dissolved O_2_ at 60 %. All other parameters were similar to those in flasks cultures.

### FAK 1384 PHA extraction and characterization

Lyophilized cell pellets were ground in a mortar and the resulting powder was extracted with chloroform for 4 h at 50 °C. The PHA-containing chloroform phase was concentrated and extracted once with water to remove residual solid particles. The organic phase was evaporated to dryness and the resulting crude extract preserved for further analyses. Purified PHA were obtained by repeated precipitations in 10 volumes of cold methanol, and analysed by Fourier transform infra red (FTIR), nuclear magnetic resonance (NMR), gas chromatography mass spectrometry (GC–MS) and differential scanning calorimetry (DSC) as previously described in Simon-Colin et al. (2008c).

## Results

### Screening for promising isolates and their origin of distribution

Out of the 760 bacterial isolates from different biological habitats (marine sediment and animals, microbial mats and films, and water column) collected around the four island groups of French Polynesia (Tuamotu, Society, Austral and Marquesas) (Table 2), 95 showed strong fluorescence under UV light when grown on Zobell media plates containing Nile red. All 95 selected isolates were confirmed PHA positive through Sudan Black staining. The results of the multiple correspondence analysis showed a very slight tendency that PHA-producing bacteria are more often found in the water column of the Society and Austral islands, respectively, on microbial mats on the Tuamotu (Additional file 1: Fig. S1). Nevertheless, there is no significant connection between the geo-ecological source and the ability of PHA-production in French Polynesia.

**Table 2:** Number of isolates per archipelago and source

Archipelagos	Microbial		Marine		Water column
	Mats	Film	Sediment	Animals	
Society	38 (2)	37 (1)	8 (−)	12 (1)	275 (48)
Tuamotu	75 (17)	47 (7)	32 (3)	26 (2)	35 (3)
Austral	10 (−)	9 (−)	2 (−)	5 (−)	53 (11)
Marquesas’	–	1 (−)	–	–	–

Number in brackets ( ) indicates isolates that were tested positive for both the Nile Red and Sudan black coloration test

### Confirmation of PHA production

70 out of these 95 promising isolates were placed in shake flask cultures for PHA production. Significant (>15 %) PHA production ability of these strains was confirmed for 25 isolates (Additional file 1: Table S1). Strain FAK 1384 appeared to be one of the most promising and interesting with a PHA content around 33 % cell dry weight (Additional file 1: Table S1). The resulting mcl-PHA polymer showed a good elasticity compared to other mcl-PHA currently studied in the lab (Moppert personal communication).

### Strain identification

On the basis of its phylogenetic analysis, strain FAK 1384 belongs to *Enterobacter* sp. (Additional file 1: Fig S2). The isolate *Enterobacter* sp. FAK 1384 has been deposited to the Collection Nationale de Culture de Microorganisms (CNCM I-4601) (Institute Pasteur, Paris, France).

### PHA characterization

**Fig. 1:**
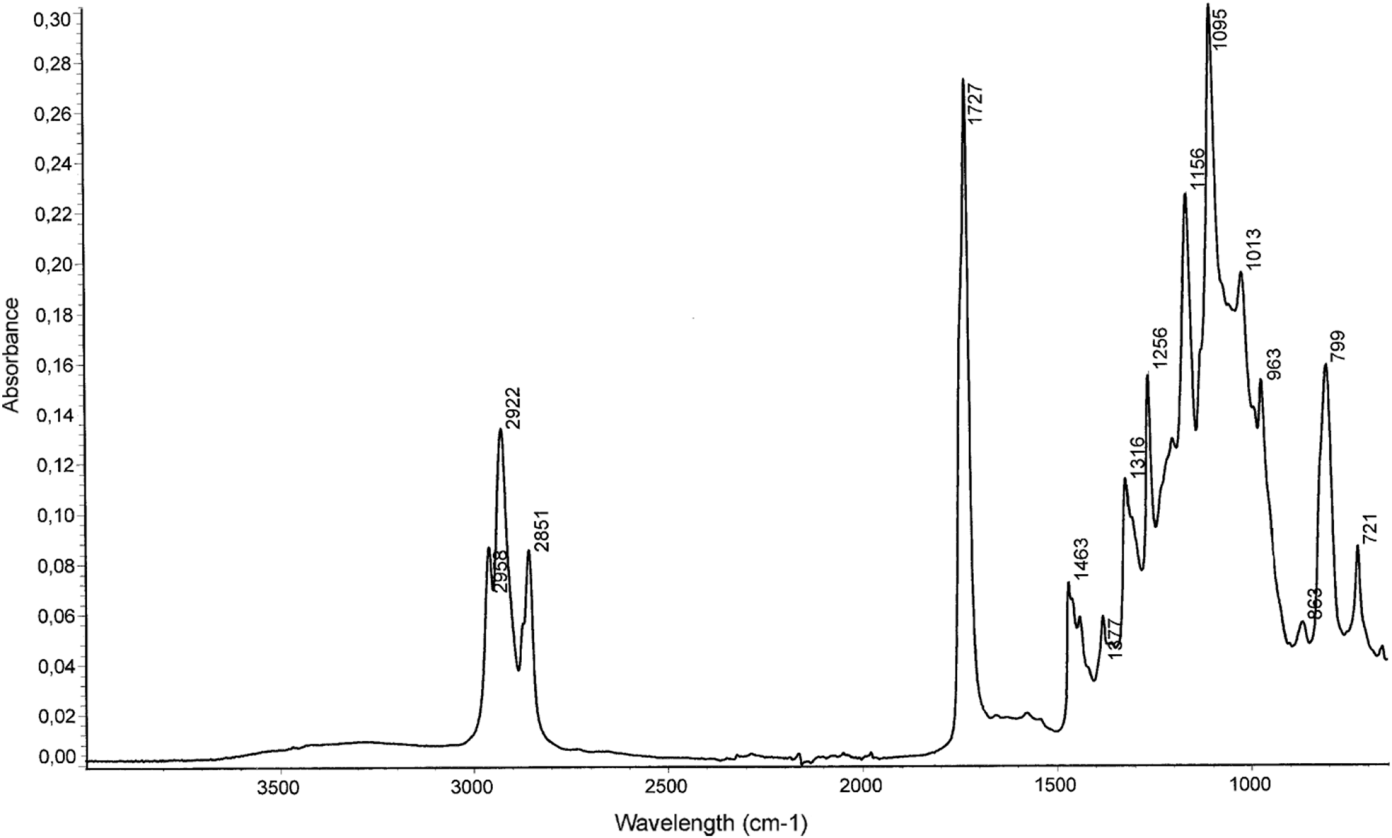
FTIR sprectrum of purified PHA produced by FAK 1384 strain grown on coprah oil

**Fig. 2:**
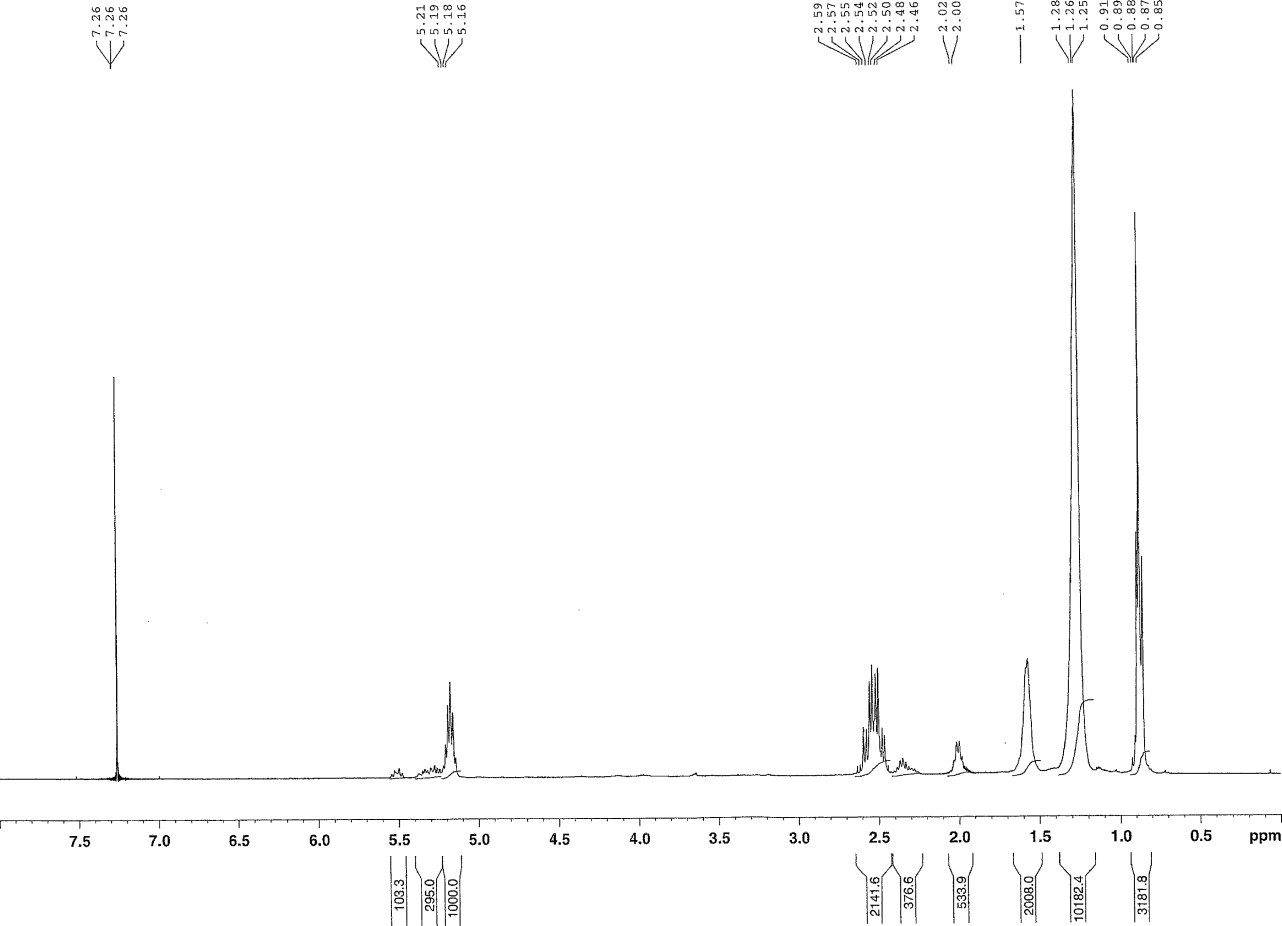
^1^H NMR sprectrum of purified PHA produced by FAK 1384 strain grown on coprah oil. The signals were assigned to methane protons (CH) at 5.2 ppm, triplet resonance at 0.89 ppm to terminal methyl group (CH_3_), multiple resonances at 2.47 ppm to the methylene protons (CH2) of C2 carbon atom. The signal at 1.58 ppm to methylene protons of the C4 carbon atom and a signal at 1.26 ppm for all other methylene hydrogens of the saturated side chains as described already in Simon-Colin et al. (2008c)

PHA synthesized by strain FAK 1384 using coprah oil as carbon substrate was purified and characterized by FTIR, DSC, NMR and GCMS as described by Simon-Colin et al. 2008c. FTIR spectra and the signals of the ^1^H NMR spectrum show the typical characters of a mcl–PHA (Figs. 1, 2). The existence of double bound can be distinguished by additional signals around 2, 2.3 and 5.3 ppm in ^1^H NMR spectrum (Simon-Colin et al. 2008c).

**Fig. 3:**
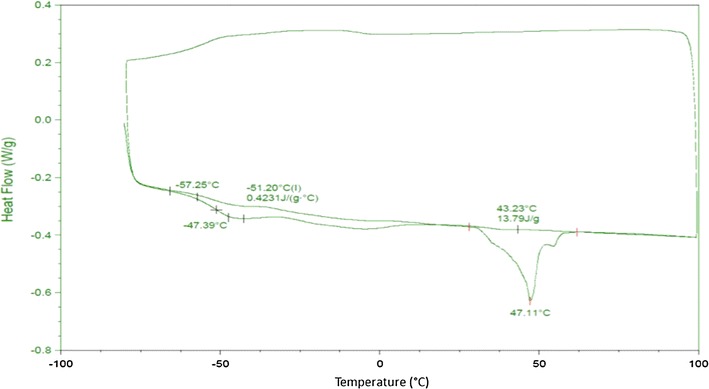
DSC analysis of purified PHA produced by FAK 1384 strain grown on coprah oil

The cristallinity index was 0.26, which indicates a quite amorphous polymer. This result was confirmed by DSC (Fig. 3) analysis showing a melting point peak (Tm) at 47 °C with a heat of fusion ΔH of 13.8 J/g, and a glass transition temperature (Tg) of −47 °C.

**Fig. 4:**
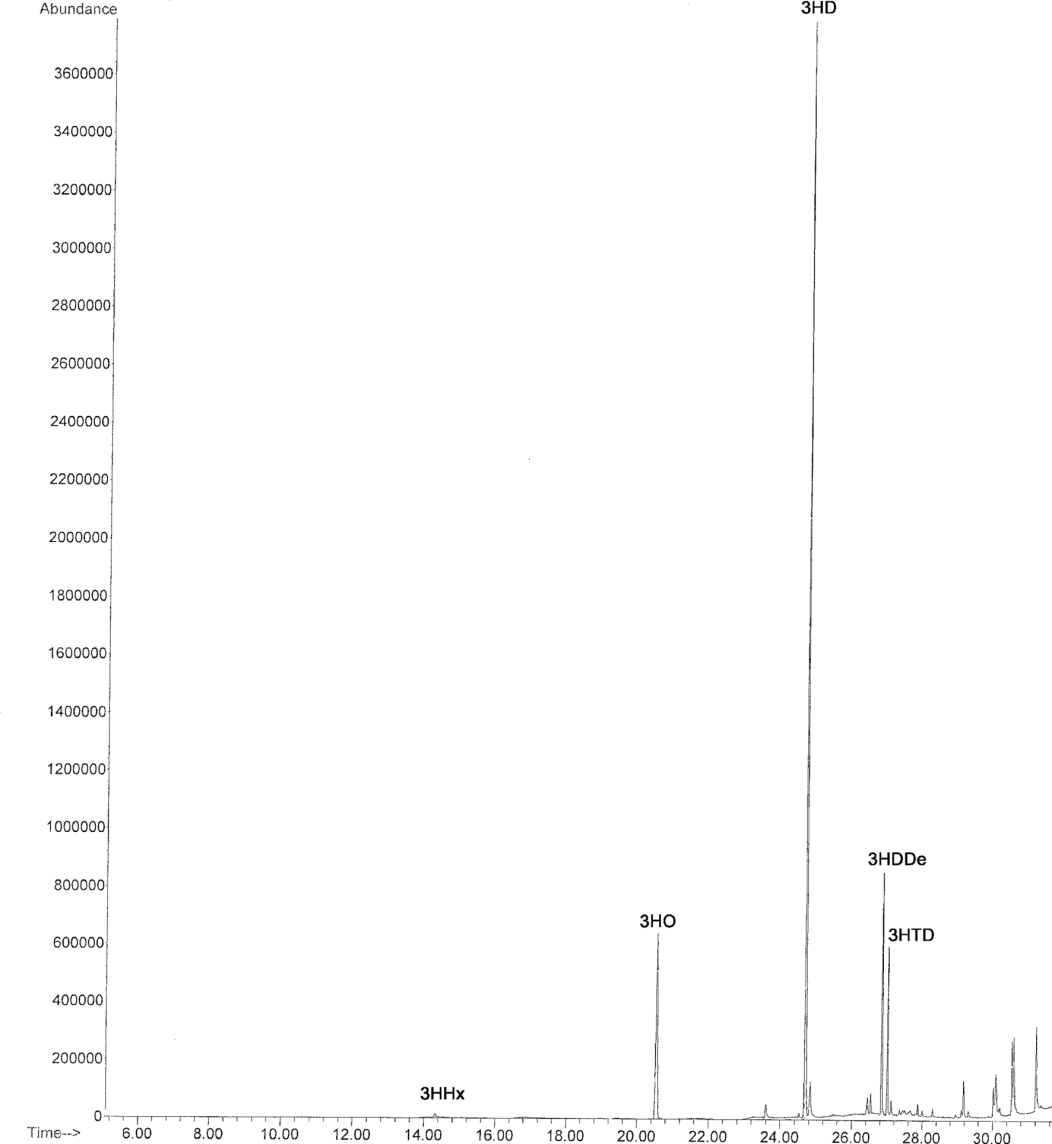
GC–MS chromatogram of TMSi methyl esters derivatives of PHA produced Fak 1384 strain

**Fig. 5:**
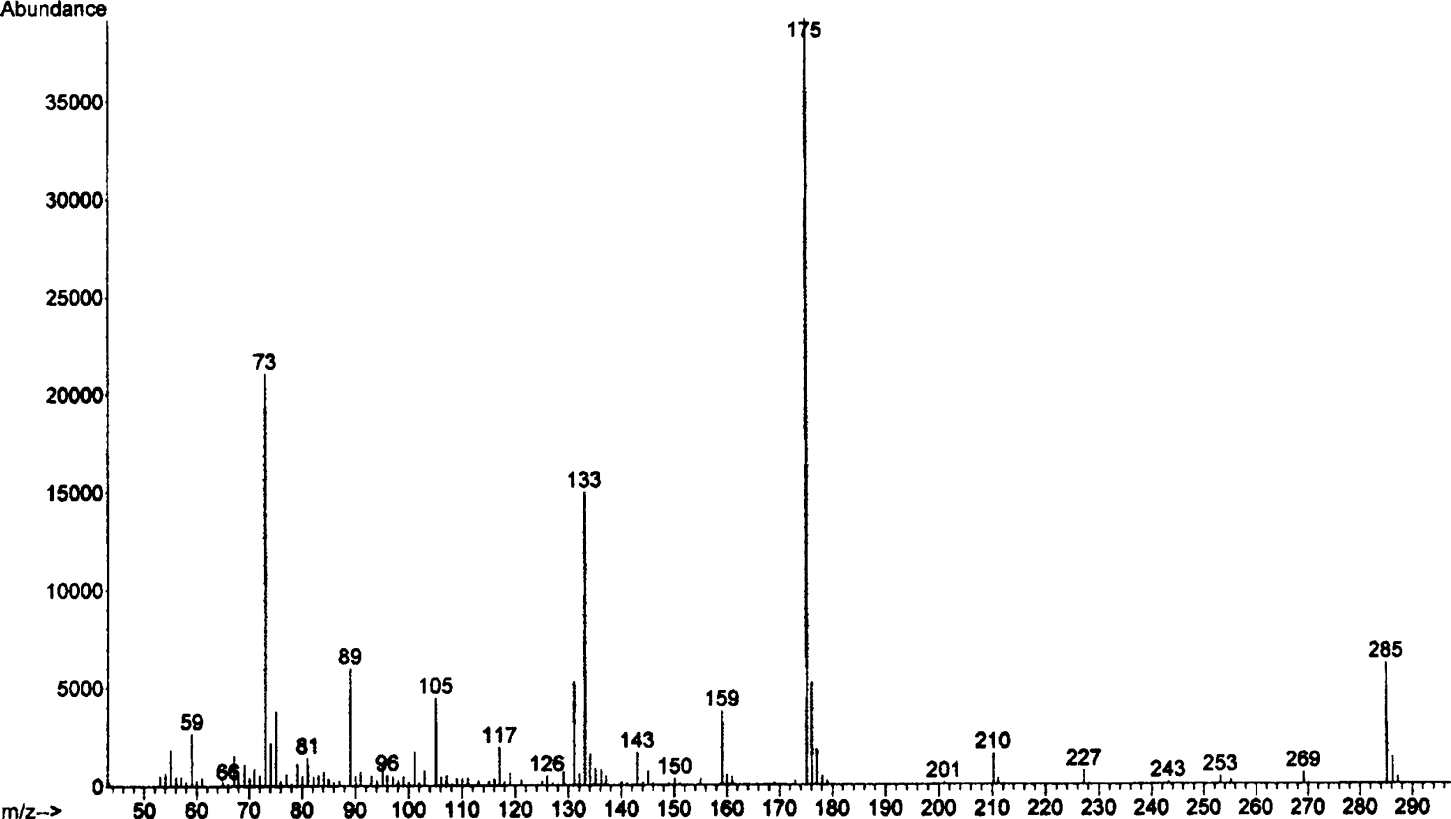
Mass spectrum of the TMSi derivatives of 3-hydroxydodecenoic acid (3HDDe) methyl esters

GC–MS analysis of TMSi methyl esters derivatives of PHA (Fig. 4) determinated an interesting monomer composition, containing 62 mol % 3-hydroxydecanoate (3HD), 18 mol % 3-hydroxyoctanoate (3HO), 12 mol % 3-hydroxydodecenoate (3HDDe), 7.6 mol % 3-hydroxydodecanoate (3HDD) and low fractions of 0.3 mol % 3-hydroxyhexanoate (3HHx) and 1.3 mol % of 3-hydroxytetradecanoate (3HTD). The presence of unsaturation can be deduced from the molecular weight of the fragment at *m*/*z* [M−15] which is 2amu less than that of the corresponding saturated monomer, and from the weak intensity ratio of the peak at *m*/*z* [M−15] to the base peak (Fig. 5).

## Discussion

12 % of the 760 bacterial isolates were confirmed for PHA positive by two coloration methods. Shake flask culturing revealed a significant level of PHA production for 25 isolates (Additional file 1: Table S1). The here presented isolated FAK1384 displayed very interesting physical parameters. The phylogenetical analysis showed that it belongs to *Enterobacter* sp. To date, according to our state of knowledge, they are only four publications dealing with PHA-producing *Enterobacter* strains (Chen et al. 2010; Samrot et al. 2011; Arumugam et al. 2014; Naheed and Jamil 2014). All of them deal with isolates from a terrestrial origin and only *Enterobacter cloacae* SU-1 produces mcl-PHA when grown on glucose or lactose (Samrot et al. 2011). Bacterial strain FAK 1384 was isolated from a shark jaw (*Carcharhinus melanopterus*) nearby Fakarava Island (Tuamotu, French Polynesia). If the isolate belongs to the bacterial community of the jaw or was taken up through the water column or with the food is not clear and needs to be investigated in the future.

However, the mcl-PHA composition is quite remarkable with a significant amount of 3-hydroxydecanoate (3HD 62 mol %), and a lower amount of 3-hydroxydodecenoate (3HDDe 12 mol %) and 3-hydroxydodecanoate (3HDD 7.6 mol %). The predomination of 3HO and 3HD is well known for various *Pseudomonas* species, independent of their carbon source (and reference within), whereas for the only further published mcl-PHA producing *Enterobacter cloacae* SU-1 grown on glucose or lactose, the main monomers are 3HO and 3HHX.

The composition of the mcl-PHA produced by FAK 1384 induces a better elasticity and a greater elongation before breaking (Hazer and Steinbuechel 2007; Rai et al. 2011). Particularly outstanding is the presence of an unsaturation in the 3-hydroxydodecenoate monomer (3HDDe). Latter represents a suitable site for chemical modifications such as chlorination or cross-linking (Arkin et al. 2000; Dufresne et al. 2001; Hazer and Steinbuechel 2007). These chemical modifications increase performances of the polymer in large application domain, e.g., coating and film manufacturing (Asrar and D’haene 1999), nucleus coating for pearl culture (Bachere et al. 2011; Guezennec et al. 2011) and in the biomedical area, such as implant material drug delivery, or tissue engineering (Hazer and Steinbuechel 2007; Chen et al. 2010; Rai et al. 2011).

In conclusion, we used low-cost and efficient staining methods to screen over 760 isolates for PHA-producing bacteria from various marine environments. PHA production was demonstrated using coprah oil as a carbon source, which is cheaper than the usual carbon sources such as glucose or lactose. We found promising PHA-producing bacteria in all tested marine environments. However, a highlight is the isolate FAK 1384, as it is a mcl-PHA contains. The extraordinary properties make this mcl-PHA a good candidate for further exploitations in specialized fields of the industry, notably, for the biomedical area or in film and coating manufacturing.

## Additional file

10.1186/s13568-015-0163-y **Fig. S1.** The multiple correspondence analysis performs a multivariate analysis, with categorical and quantitative variables. Two individuals are close to each other if they shared the same traits (variables). The green triangles show the presence or absence of PHA between the different associations. Isolates are shown in blue. MarSed: Marine sediment, MicroMat: Microbial mats, MarFilm: Marine film and MarAni: Marine animals. **Fig. S2**: The maximum-likelihood reconstruction of the 16S nuclear ribosomal DNA genotypes of *Enterobacter* shows the phylogenetic position of FAK1384 within the genera *Enterobacter*. Accession numbers are put in brackets. Node numbers indicate percentage bootstrap support from 500 replicates. Nodes without bootstrap values were supported by less than 75 % of the replicates. **Table S1**: 25 out of the 70 tested isolates show a significant PHA production ability (> 15 %).
