# Supplementary material for: Discovery of a mcl-PHA with unexpected biotechnical properties: the marine environment of French Polynesia as a source for PHA-producing bacteria
Source: AMB Express. 2015 Nov 25;5:74. doi: 10.1186/s13568-015-0163-y (PMC4659796; doi:10.1186/s13568-015-0163-y)
Supplement: Supplementary file 1 — 10.1186/s13568-015-0163-y Fig. S1. The multiple correspondence analysis performs a multivariate analysis, with categorical and quantitative variables. Two individuals are close to each other if they shared the same traits (variables). The green triangles show the presence or absence of PHA between the different associations. Isolates are shown in blue. MarSed: Marine sediment, MicroMat: Microbial mats, MarFilm: Marine film and MarAni: Marine animals. Fig. S2: The maximum-likelihood reconstruction of the 16S nuclear ribosomal DNA genotypes of Enterobacter shows the phylogenetic position of FAK1384 within the genera Enterobacter. Accession numbers are put in brackets. Node numbers indicate percentage bootstrap support from 500 replicates. Nodes without bootstrap values were supported by less than 75 % of the replicates. Table S1: 25 out of the 70 tested isolates show a significant PHA production ability (> 15 %). [file 13568_2015_163_MOESM1_ESM.pdf]

Discovery of a mcl-PHA with unexpected biotechnical properties: the marine environment of French Polynesia as a source for PHA-producing bacteria

P. Wecker[1 $], X. Moppert[2*], C. Simon-Colin[3*], B. Costa[2] and V. Berteaux-Lecellier[1]

1 USR3278 CNRS-EPHE-UPVD CRIOBE, BP1013, Papetoai, Moorea, French Polynesia

2 Pacific Biotech SAS, BP140289, Arue, Tahiti, French Polynesia,

3 Ifremer, Laboratoire de Microbiologie des Environnements Extrêmes, UMR6197, M2E, Plouzané, France

$ Corresponding author: wecker.pat@gmail.com, Tel: +689 40 56 13 45, Fax: +689 50 56 28 15

* Contributed equally

Fig S1 in the additional file1: The multiple correspondence analysis performs a multivariate analysis, with categorical and quantitative variables. Two individuals are close to each other if they shared the same traits (variables). The green triangles present the presence or absence of PHA between the different associations. Isolates a shown in blue. MarSed: Marine sediment, MicroMat: Microbial mats, MarFilm: Marine film and MarAni: Marine animals.

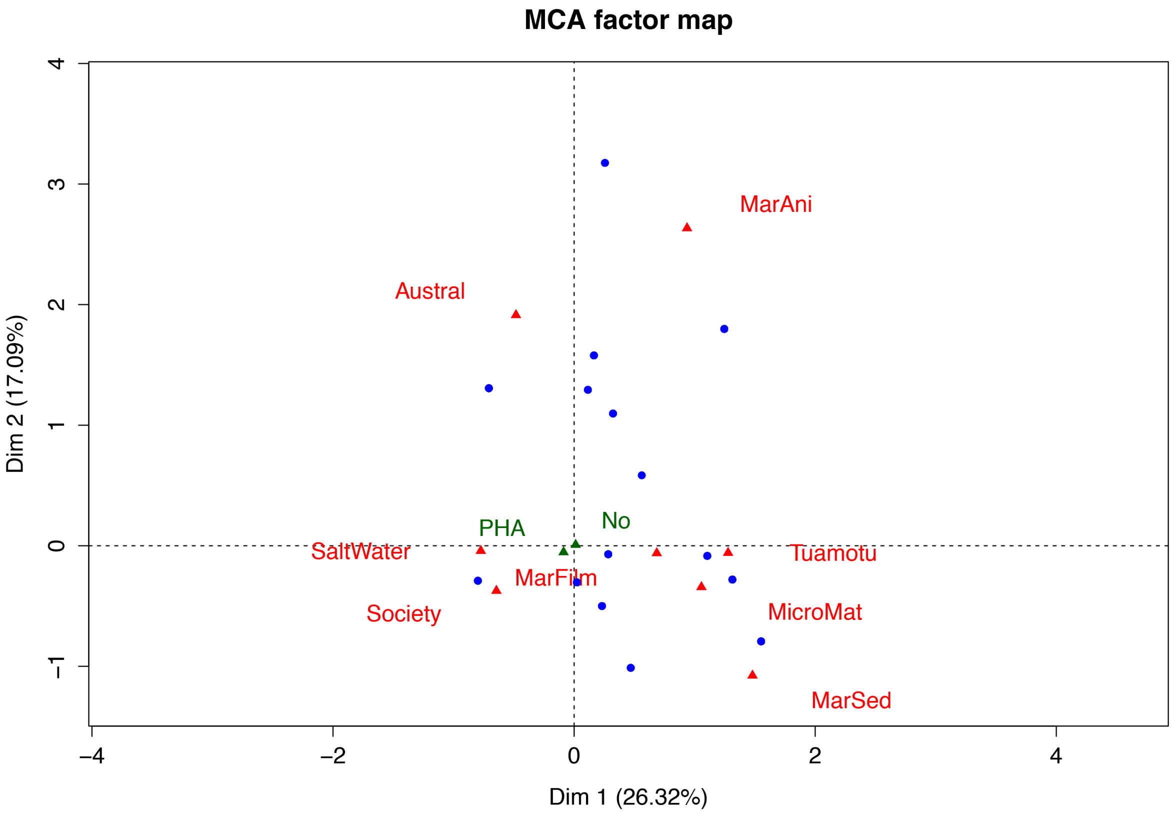

Discovery of a mcl-PHA with unexpected biotechnical properties: the marine environment of French Polynesia as a source for PHA-producing bacteria

P. Wecker[1 $], X. Moppert[2*], C. Simon-Colin[3*], B. Costa[2] and V. Berteaux-Lecellier[1]

Table S1 in the additional file: 25 out of the 70 tested isolates show a significant PHA production ability (>15%)

Strain	Biomass (g/L)	PHA (g/L)	Content (%)	Taxonomy	Source
RA 42	3,2	1,0	30	*Firmicutes*	Microbial mat
RA 58	4,7	0,7	15	*γ-proteobacteria*	Microbial mat
RP75	4,4	1,0	24	*Firmicutes*	Water column
RP 84	3,2	0,7	21	*γ-proteobacteria*	Water column
RP 106	2,5	0,6	23	*γ-proteobacteria*	Water column
RP 123	2,0	0,6	30	*Firmicutes*	Water column
RA 136	2,0	0,4	22	*γ-proteobacteria*	Microbial mat
RA 137	2,0	0,3	17	*γ-proteobacteria*	Microbial mat
RA 146	5,2	1,3	24	*γ-proteobacteria*	Microbial mat
MO 177	1,7	0,4	21	*γ-proteobacteria*	Water column
MO 188	1,7	0,4	22	*γ-proteobacteria*	Water column
MO 277	5,6	1,1	20	*γ-proteobacteria*	Water column
TH 395	3,6	0,9	26	*γ-proteobacteria*	Water column
TA 465	1,1	0,2	16	*γ-proteobacteria*	Water column
TA 510	2,6	0,6	23	*γ-proteobacteria*	Water column
TIK 588	3,1	0,5	15	*γ-proteobacteria*	Marine sediment
TIK 593	6,2	1,4	22	*Firmicutes*	Microbial mat
TIK 594	2,6	0,4	16	*γ-proteobacteria*	Microbial film
TIK 599	3,0	0,4	14	*γ-proteobacteria*	Water column
MO 727	6,7	1,8	27	*α-proteobacteria*	Water column
MO 728	3,7	1,1	30	*γ-proteobacteria*	Water column
MO 729	1,8	0,3	15	*α-proteobacteria*	Water column
MO 730	2,3	0,4	19	*α-proteobacteria*	Water column
FAK 1350	4,5	0,8	19	*γ-proteobacteria*	Marine sediment
FAK 1384	3,1	1,0	33	*γ-proteobacteria*	Marine animal

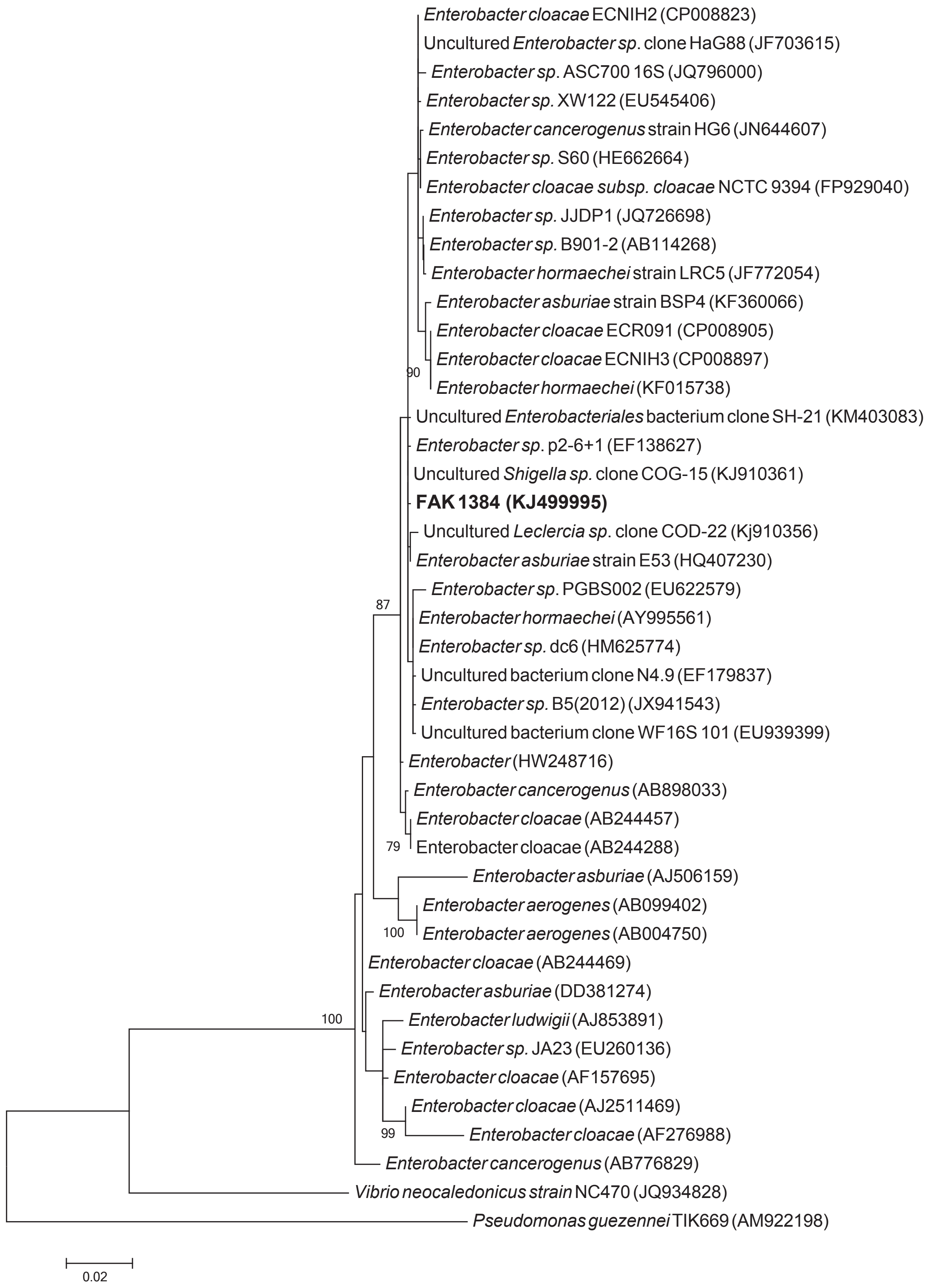

Fig S2 in the additional file: The maximum-likelihood reconstruction of the 16S nuclear ribosomal DNA genotypes of *Enterobacter* shows the phylogenetic position of FAK1384 within the genera *Enterobacter*. Accession numbers are put in brackets. Node numbers indicate percentage bootstrap support from 500 replicates. Nodes without bootstrap values were supported by less than 75 % of the replicates
